# Homoeologs: What Are They and How Do We Infer Them?

**DOI:** 10.1016/j.tplants.2016.02.005

**Published:** 2016-07

**Authors:** Natasha M. Glover, Henning Redestig, Christophe Dessimoz

**Affiliations:** 1 Bayer CropScience NV, Technologiepark 38, 9052 Gent, Belgium; 2 University College London, Gower Street, London WC1E 6BT, UK; 3 University of Lausanne, Biophore, 1015 Lausanne, Switzerland; 4 Swiss Institute of Bioinformatics, Biophore, 1015 Lausanne, Switzerland

**Keywords:** homoeology, polyploidy, homology, positional homoeology

## Abstract

The evolutionary history of nearly all flowering plants includes a polyploidization event. Homologous genes resulting from allopolyploidy are commonly referred to as ‘homoeologs’, although this term has not always been used precisely or consistently in the literature. With several allopolyploid genome sequencing projects under way, there is a pressing need for computational methods for homoeology inference. Here we review the definition of homoeology in historical and modern contexts and propose a precise and testable definition highlighting the connection between homoeologs and orthologs. In the second part, we survey experimental and computational methods of homoeolog inference, considering the strengths and limitations of each approach. Establishing a precise and evolutionarily meaningful definition of homoeology is essential for understanding the evolutionary consequences of polyploidization.

## Trends

The term homoeology has been used inconsistently in historical and modern contexts.

Homoeologs are pairs of genes that originated by speciation and were brought back together in the same genome by allopolyploidization.

Homoeologs are not necessarily one-to-one or positionally conserved.

Evolution-based computational methods have emerged to infer homoeologs from sequencing data.

## Polyploidization and Homoeology

Many plants – and virtually all angiosperms – have undergone at least one round of polyploidization in their evolutionary history 1, 2, 3. In particular, numerous important crop species, such as *Arachis hypogaea* (peanut), *Avena sativa* (oat), *Brassica juncea* (mustard greens), *Brassica napus* (rapeseed), *Coffea arabica* (coffee), *Gossypium hirsutum* (cotton), *Mangifera indica* (mango), *Nicotiana tabacum* (tobacco), *Prunus cerasus* (cherry), *Triticum turgidum* (durum wheat), and *Triticum aestivum* (bread wheat), exhibit **allopolyploidy** (see Glossary), a type of whole-genome duplication via hybridization followed by genome doubling [4]. This hybridization usually occurs between two related species, thus merging the genomic content from two divergent species into one (Box 1).

Allopolyploidization has been studied since at least the early 1900s. Some of the first investigations were about chromosome numbers and pairing patterns of hybrid species 5, 6. The term **homoeologous** was coined to distinguish chromosomes that pair readily during meiosis from those that pair only occasionally during meiosis [7]. However, the definition of homoeology has varied and at times been used inconsistently.

Homoeology has been broadly used to denote the relationship between ‘corresponding’ genes or chromosomes derived from different species in an allopolyploid. Accurately identifying homoeologs is key to studying the genetic consequences of polyploidization; knowing the evolutionary correspondence between genes across **subgenomes** allows us to more accurately estimate gene gain or loss after polyploidization (reviewed in 8, 9) and to study the major structural rearrangements or conservation between homoeologous chromosomes. Additionally, we can study the functional divergence of homoeologs on polyploidization, particularly in terms of expression (reviewed in 2, 8, 10, 11, 12, 13), epigenetic patterns (reviewed in 8, 12), alternative splicing [14], and diploidization (reviewed in [8]). From a crop improvement viewpoint, identifying homoeologs that may have been functionally conserved is important for elucidating or engineering the genetic basis for traits of interest 15, 16.

This high interest in the genetic and evolutionary consequences of polyploidization has driven the development of several methods for homoeolog inference. However, because of their highly redundant nature polyploid genomes have been notoriously challenging to sequence and assemble [17]. Recent breakthroughs in sequencing and assembly methods suggest that we are finally overcoming this hurdle 18, 19, 20 and as increasing numbers of polyploid genomes are sequenced there will be a growing interest in homoeology inference. Thus, it is necessary to establish a common framework.

Here we examine the current and common definitions of homoeology and point out imprecise usage in the literature, from historical definitions to modern understandings. We advocate a precise and evolutionarily meaningful definition of homoeology and connect homoeology and **orthology** inference. We then review homoeolog inference methods and discuss advantages and disadvantages of each approach.

## What Are Homoeologs?

### Historical Definitions and Modern (Mis)Understandings

It is first important to make the distinction between **homology** and homoeology. The prefix ‘homo-’ comes from the Latin (and ancient Greek) word for ‘same’, whereas the prefix ‘homoeo’ means ‘similar to’ [21]. Homoeology has alternatively been spelled as ‘homeology’ (Box 2). Both terms have a history of varied and, at times, inconsistent usage in different fields, but in biology it is now generally accepted that homology indicates ‘common ancestry’; by contrast, ‘homoeology’ is more ambiguous.

The term homoeologous was first used in a cytogenetics study of allopolyploid wheat, where Huskins (1931) defined it as ‘phylogenetically similar but not strictly homologous chromosomes’ in a hybrid. Huskins goes on to explain further:

To distinguish between chromosomes which come within the commonly accepted meaning of the term homologous and those which are, as evidenced by their pairing behavior, similar only in part, the latter might be referred to as homœologous chromosomes, signifying similarity but not identity…This term would include chromosomes of different ‘genomes’ which pair occasionally in allopolyploids, often causing the appearance of mutant or aberrant forms, and also, as a corollary, chromosomes which pair irregularly in many interspecific hybrids. [7]

Two decades later, in the 1949 *Dictionary of Genetics*, R.L. Knight defines homoeologous chromosomes as ‘chromosomes that are homologous in parts of their length’ [22].

Thus, in its historical context, a pair of homoeologous chromosomes is thought of as being similar but exhibiting only infrequent pairing during meiosis. In a survey of 93 studies of autopolyploids and 78 studies of allopolyploids, multivalent pairing (pairing between more than two chromosomes) on average occurred more in autopolyploids than in allopolyploids (∼29% vs 8%) [23]. Although chromosome pairing patterns give a good indication of homology type, this should not be used as a criterion (Box 1).

Over the years, the definition of homoeology has evolved and diverged to have different usages depending on the scientific field of study or topic. The term homoeologous can mean different things and may not be as simple as ‘genes duplicated by polyploidy’ [24]. Table 1 highlights the differences between the different definitions of homoeology depending on the context in which it is used. The variation among definitions depends on the level of biological analysis: at the chromosome, gene, or sequence level.

Even in modern evolutionary biology contexts, the term homoeolog has been used inconsistently. For instance, some have used it not just in the context of allopolyploids but to relate duplicates created by **autopolyploidy** as well (for example, 25, 26). This is, however, at odds with the original description of homoeologs as belonging to an allopolyploid genome [7]. There are biological differences between genes that arise due to speciation versus duplication [27] and thus also, conceivably, between allo- versus autopolyploids. Autopolyploids by definition are created by genome doubling, with an exact copy of the genome formed. By contrast, allopolyploids are formed by the merger of closely related species that have already started to diverge. Although still poorly understood, these fundamental differences could have significant effects on the genome of the polyploid. Hybridization can induce a ‘genome shock’ prompting epigenetic or expression changes that might not be present with strictly genome doubling *per se* 8, 28, 29, 30. The functional consequences of genes duplicated by allo- vs autopolyploidy still needs to be investigated, which is why a clear distinction of terminology between the two is important. Furthermore, this usage of homoeolog overlaps with another term – **ohnologs** – used to denote genes resulting from whole-genome duplication [31].

The term homoeolog has even been used to refer to similar chromosomal regions in different species 32, 33, 34. Although closely related species do have similar chromosomes and gene content, this latter usage is unorthodox: the term homoeolog has been overwhelmingly used to denote relationships within polyploids, and therefore within a single species rather than between closely related species. A cross-species definition of homoeology is also redundant with that of orthology.

### A Unifying, Evolutionarily Precise Definition of Homoeology

**Figure 1:**
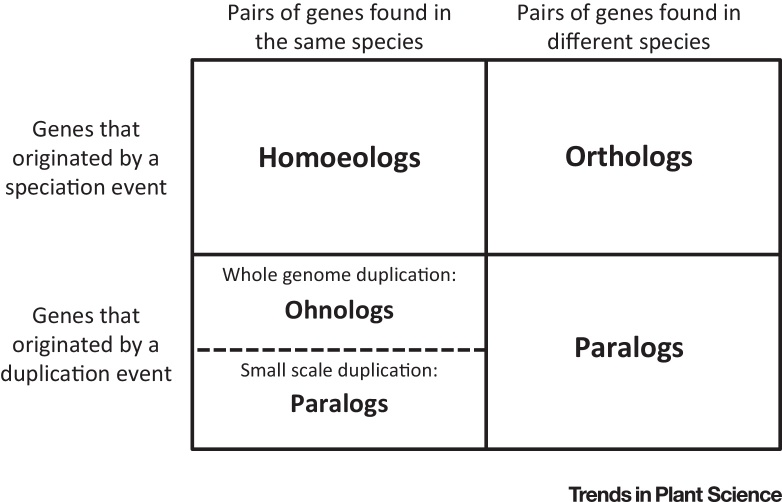
Subtypes of Homologous Genes (Genes of Common Ancestry). As the table shows, the definition of ‘homoeologs’ we recommend – genes that originated by speciation and that were subsequently brought back in a single genome through allopolyploidization – complements well other homology subtypes commonly used in evolutionary biology. In particular, the table highlights the parallels between homoeologs and orthologs and between homoeologs and ohnologs.

Consequently, there is a need for a unifying, evolutionarily precise definition of homoeology, formulated in terms of the key events that gave rise to the genes in question. The ideal definition should be as consistent as possible with the widespread usage of the term and should complement the other ‘-log’ terms, which have served the community well. We define homoeologs as pairs of genes or chromosomes in the same species that originated by speciation and were brought back together in the same genome by allopolyploidization. Figure 1 depicts how this definition complements the other ‘log’ terms. In particular, the analogy between homoeologs and orthologs implies that homoeologs can be thought of as orthologs between subgenomes of an allopolyploid [35].

Note that the term ‘**paleolog**’ is sometimes used to denote ancient polyploidization events. The term is convenient for plants such as soybean where the polyploidization event occurred more than a few million years ago and where it is unknown whether these were auto- or allopolyploidization events [36].

### Implications of the Definition for Positional Conservation and Relationship Cardinality

Because of the analogy between homoeology and orthology, homoeologs are under the same common misconceptions that afflict orthologs: the notion that homoeologs necessarily in a one-to-one relationship or that they have remained strictly in their ancestral positions since speciation.

**Figure 2:**
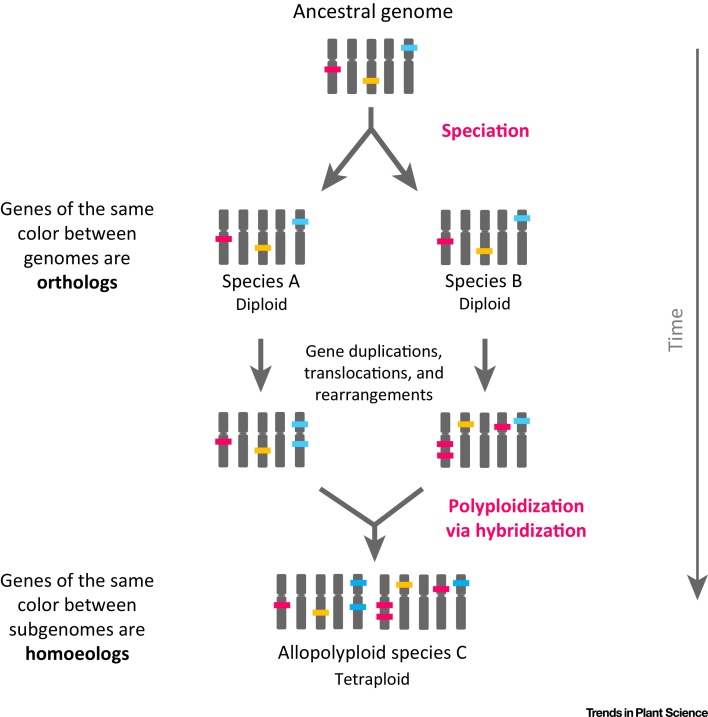
Evolutionary History of an Allopolyploid. An ancestral genome undergoes a speciation event, resulting in two diploid species. The genes, which descended from a common gene in the ancestor, are orthologs. Evolution occurs after speciation, including structural rearrangements, gene duplications, and gene movement. On polyploidization, genes that were once orthologs are now homoeologs. Homoeologous relationships can be one-to-one, one-to-many, or many-to-many depending on the number of duplications since speciation of the progenitors.

Since homoeology is characterized by an initial speciation event, once the progenitor species of the future allopolyploid begin to diverge, the corresponding genes in each new species that descended from a common ancestral gene start diverging in sequence (Figure 2). The sequence divergence will depend on the time since the progenitor divergence and other factors (the same factors that contribute to ortholog divergence such as selection pressure, duplication events, and others). In addition to genic sequence divergence, other scale evolutionary events may occur, including single-gene duplications, deletions, and rearrangements.

As a consequence, orthologous relationships are not necessarily one-to-one between species and may exist in one-to-many or many-to-many relationships, especially among highly duplicated plant genomes [37]. The same is true for homoeologous relationships. Depending on the duplication (and loss) rate since the divergence of the progenitor species, there may be more than one homoeologous copy of a given gene per subgenome (Figure 2).

In many plant species, a high degree of collinearity, or conservation of gene order [38], has been observed between homoeologous chromosomes in polyploids. Genes tend to stay in their ancestral position since divergence, leading to the concept of positional orthology [39] and, analogously in allopolyploids, of **positional homoeology**. However, there may be rearrangement of homoeologs via single-gene duplication/translocation either before or after polyploidization, going against the widespread notion that homoeologous genes are always positional (i.e., have remained in their ancestral location), as stated for example in [25].

Although we can expect that most homoeologs remain positionally conserved and in a one-to-one relationship after polyploidization, these are only a subset of the homoeologs. The frequency of homoeolog duplication may be significantly underestimated in some species due to use of the best bidirectional hit (BBH) – an approach inherently limited to inferring one-to-one relationships 40, 41.

## How to Infer Homoeologs

In general, homoeology inference reduces to identifying similar (and therefore likely homologous) genes within a polyploid genome and inferring whether pairs of homologs started diverging from one another through speciation, in which case they are homoeologs (and usually located on different subgenomes), or through duplication, in which case they are **paralogs** (and usually located on the same subgenome). The methods for doing so have changed over time with advances in technology, from low-throughput laboratory techniques to high-throughput computational ones. In this section we survey these techniques and highlight their relative strengths and limitations.

### Wet Lab Techniques Based on Probe Hybridization or PCR Amplification

Although whole-genome sequencing has become commonplace thanks to next-generation sequencing (NGS) techniques, many species do not yet have a fully sequenced reference genome. Techniques used to isolate homoeologous genes from polyploid species before NGS were based on hybridization, using a probe or primer as a template to retrieve the homoeologs of interest. However, due to the high sequence similarity of homoeologs as well as paralogs, one would obtain a mixture of DNA molecules representing homoeologous and paralogous copies, which then needed to be separated.

One method of separating homoeologous copies from each other in a pool of highly similar DNA molecules is by using the mixture of homoeologs obtained from PCR to transform into bacteria, resulting in only a single copy of either homoeolog in each bacterial colony. Colonies can then be isolated, sequenced, and assigned to subgenomes by using knowledge from diploid progenitor species, specifically differential (sub)genome restriction patterns [42]. Note that the true progenitors may no longer exist in nature and that the term ‘progenitor’ may refer to their extant, unhybridized descendant or close relative [43].

Another way of separating homoeologous sequences makes use of restriction-digested DNA followed by size fractionation on a gel [44]. Minor differences among homoeologous copies can be expected to result in sequence differences at restriction sites and thus digestion cuts homoeologous copies into different sizes. This is followed by isolating the DNA from the separated bands and then amplifying these homoeologous copies by cloning. Alternatively, isolated homoeologs can be obtained using PCR primers to produce a mixture of homoeologous copies, and after size fractionation the same primers can be used to amplify individual homoeolog copies [44].

The above techniques are all performed on a gene-by-gene basis with molecular methods and therefore are small scale and relatively time-consuming and laborious. A more recent and larger-scale technique to separate homoeologs, based on hybridization of genomic DNA to an array, is able to target hundreds or thousands of genes at a time, each individually spotted on the array. Salmon *et al.* [45] used this technique to capture homoeologous pairs in *G. hirsutum*. After hybridization, the probes on the chip, enriched for homoeologous pairs, were then sequenced with NGS. Homoeologs could be distinguished by sequence polymorphisms between them.

These experimental techniques have several limitations. First, they are appropriate for studies focusing on a small number of genes but scale poorly to entire genomes. Additionally, they all require prior sequence information for the gene of interest. If cDNA is used as the starting point, one can combine homoeolog inference with differential expression studies. However, this works only for genes that are expressed in the particular condition from which the cDNA library was made. Homoeologs are assigned to a subgenome by comparing the individual homoeolog sequences from the polyploid to their orthologous counterparts in the diploid progenitors. Therefore, these experiments need to be performed on the progenitor species as well, which may not always be readily available. Finally, it can be difficult to distinguish homoeologous from paralogous sequences, as the degree of sequence divergence between the two can be slight and thus not result in a clear difference in hybridization pattern. Thus, these techniques do not perform well on large gene families.

### Comparative Mapping and Positional Homoeology

Before the era of whole-genome sequencing, molecular markers were used to detect synteny and collinearity between chromosomes. However, molecular mapping is more complicated in a polyploid than a diploid, as there needs to be sufficient allele polymorphism to distinguish among the different homoeologs. Several techniques exist to circumvent this problem by **comparative mapping** in diploid relatives or by using aneuploid lines [46]. Many studies have been published using mapping to identify homoeologous relationships between chromosomes or genes in several allopolyploids, including *Gossypium* (cotton) [47], *B. napus* (rapeseed) [48], *A. hypogaea* (peanut) [49], and *T. aestivum* (wheat) 50, 51, 52, 53, 54. Wheat researchers played a major role in popularizing the term homoeology in the 1990s, with many molecular mapping papers showing the collinearity between wheat homoeologous chromosomes.

Although conservation of position in the genome can be used as another layer of evidence above sequence similarity to infer homoeology, there are several inherent problems with homoeology inference based solely on this approach. Mapping homoeologs is possible only if the molecular markers are able to distinguish sequence polymorphisms between homoeologs. Additionally, conservation of relative genomic location in itself is not a requirement for homoeology, which depends only on the type of event that gave rise to the sequences. Due to potential duplications, chromosomal rearrangements, or other events leading to gene movement 55, 56, 57, relying on positional conservation to infer homoeology may lead to a substantial fraction of missed homoeologous relationships and introduce a bias. Like orthologs or paralogs, positional and non-positional homoeologs could differ in their biological characteristics. For example, orthologous genes maintained in the same position have slower evolution rates, are less likely to undergo positive selection, and are more likely to have a conserved function 58, 59, 60, 61, 62. Additionally, positional orthologs have been shown to maintain a higher expression level and breadth compared with non-positional orthologs [63]. Paralogs that have inserted into distant regions of the genome tend to have a more divergent DNA methylation pattern and expression than tandem duplicates 64, 65.

### Similarity-Based Computational Techniques

High-throughput sequencing allows fast and affordable production of genome-wide sequence information, making it possible to identify similar regions and infer homoeology computationally at a genome-wide scale. However, despite rapid improvements in sequencing technology it remains a challenge to obtain a high-quality, fully assembled reference genome sequence for many plant species [66]. This is mainly because of their large, complex genomes, which are highly repetitive due to duplication and transposon activity [17]. With entire chromosomes in multiple copies, this difficulty is compounded in polyploid genomes. Because of these issues, most polyploid plant genome sequences remain in a draft, highly fragmented state, usually comprising small contigs harboring only a few genes [17].

The identification of homoeologs thus first requires assembling short sequences (e.g., expressed sequence tags or, increasingly, NGS reads) at low stringency followed by homoeolog discrimination based on sequence polymorphisms between the reads. For example, Udall *et al.* assembled ESTs from allotetraploid cotton and the two diploid progenitors. Most assembled contigs contained four copies: two orthologs from the progenitors and one from each of the homoeologs. They then assigned the homoeolog ESTs to their appropriate subgenome based on sequence comparison with the progenitors [67].

In another example [68], homoeologs were distinguished in hexaploid wheat by first assembling, at a relatively low stringency, transcriptome NGS reads into clusters of sequences containing homoeologs and close paralogs. The second step was to reassemble each cluster separately using a more stringent assembler to separate homoeologs.

After discriminating between homoeologous genes, it is generally necessary to map the reads back to the progenitor species to infer to which subgenome they belong. For example, Akama *et al.* [69] sequenced and *de novo* assembled both *Arabidopsis halleri* and *Arabidopsis lyrata* (progenitors of the allotetraploid *Arabidopsis kamchatica*). They identified homoeologs by aligning the allotetraploid reads to both the *A. halleri* and *A. lyrata* genomes and considered high-scoring alignments as homoeologs. A similar technique was performed in hexaploid wheat taking advantage of the recently sequenced diploid progenitors *Triticum urartu* and *Aegilops tauschii* [70]. Another method of separating contigs into individual homoeolog copies employs the strategy of ‘post-assembly phasing’ using remapped reads, which detects polymorphisms in reads and determines whether they were inherited together [71].

Provided that the progenitors’ genomes are known and well separated, techniques based on short reads and sequence polymorphisms to infer homoeologs can be effective. Because they tend to be based on RNA-seq reads, one can simultaneously quantify their expression. However, there will be false negatives if one or both of the homoeologs is unexpressed. Also, it can be costly to first sequence the progenitor species. Another disadvantage is that, again, these methods do not establish one-to-many or many-to-many relationships. Additionally, as with experimental hybridization methods, it can be difficult to distinguish homoeologs from paralogs.

### Evolution-Based Computational Techniques

We indicated above that homoeologs should be defined as pairs of genes within an allopolyploid that originated by speciation and were reunited by hybridization. Thus, fundamentally, the relationship between homoeologs is based on evolutionary relationships rather than sequence similarity. Furthermore, the parallel between homoeologs and orthologs suggests the possibility of repurposing orthology inference methods – a relatively mature area of research with many well-established computational methods [72]. These methods, which all work at the genome-wide scale, are divided into phylogenetic-tree-based (which infer speciation and duplication nodes on gene trees) and graph-based (which infer the evolutionarily closest genes between species without explicitly reconstructing trees).

Methods based on phylogenetic trees use the process of gene/species tree reconciliation, which determines whether each internal node of a given gene tree is a speciation or duplication node using the phylogeny of the species tree. With this information one can determine whether any two genes are related through orthology or paralogy; pairs of genes that coalesce at a speciation node are orthologs, whereas pairs of genes that coalesce at a duplication node are paralogs [72]. To our knowledge, the only phylogenetic tree-based homoeology inference approach taken so far is that of Ensembl Genomes, which has repurposed their Compara phylogenetic tree-based pipeline [73] to distinguish orthologs, paralogs, and homoeologs in wheat [74]. This is achieved by treating each subgenome as a different species, running their usual orthology pipeline, and finally relabeling orthologs inferred among subgenomes as homoeologs. This information is found in the ‘location-based display’ on their website under ‘Polyploid view’ (http://plants.ensembl.org/Triticum_aestivum/Info/Index).

In general, graph-based orthology methods comprise inferring and clustering pairs of orthologs based on sequence similarity [72]. Graph-based orthology methods have also been adapted to infer homoeologs. One of the simplest and most widely used methods of ortholog detection is by finding BBHs between pairs of genomes [75]. This method uses BLAST [76] or another sequence alignment algorithm to find the set of reciprocally highest-scoring pairs of genes between two genomes. Such an approach was used to infer homoeologs between the subgenomes of hexaploid wheat, identifying triplets of best bidirectional protein hits between subgenomes [41]. However, the BBH method has inherent drawbacks. By identifying only the ‘best’ pair, it cannot identify one-to-many or many-to-many homoeology. This is particularly problematic for highly duplicated plant genomes [77]. As a result, BBH between subgenomes will at best infer a subset of the homoeologous relationships, thereby yielding false-negatives. Additionally, differential gene loss among the subgenomes can cause erroneous inference of paralogs as homoeologs [78]. Finally, using alignment scores is suboptimal in the presence of many fragmentary genes and sequencing errors [79].

Another graph-based homoeolog inference approach to analyze the wheat genome was performed in the Orthologous Matrix (OMA) database – a method and resource for inferring different types of homologous relationships between fully sequenced genomes [35]. This technique identifies mutually closest homologs based on evolutionary distance while considering the possibility of differential gene loss or many-to-many relationships [80]. Again, the application of the orthology inference pipeline was achieved by treating each subgenome as a different species, running the standard pipeline, and, finally, calling orthologs between subgenomes homoeologs. Compared with the BBH approach, the OMA algorithm has the advantages of considering many-to-many homoeology, identifying differential gene losses, and relying on evolutionary distances rather than alignment score.

The main issue limiting the use of repurposed orthology methods such as Ensembl Compara and OMA is the requirement for *a priori* delineation of the subgenomes. If there have been rearrangements across subgenomes since hybridization of the progenitors occurred, this will cause errors in homoeolog inference because subgenomes can no longer be straightforwardly treated as individual species. Another problem with both similarity- and evolution-based techniques is that they are highly dependent on the quality of sequence assembly and annotation used to infer homoeologs (Box 3).

## Concluding Remarks

Polyploid species are widespread throughout the plant kingdom. There is much interest in polyploidy and accurately identifying homoeologs allows us to better study the genetic and evolutionary consequences on genomes of polyploids. Many exciting findings have been published recently that provide insights into the structural and functional divergence of homoeologs and the chromosomes they reside on 3, 9, 81, 82. As a result, polyploidy has emerged as potentially a major mechanism of adaptation to environmental stresses 9, 83, 84, 85.

The term homoeologous was first used in 1931 to describe chromosomes related by allopolyploidy. Since then, the definition has changed over the years and now suffers from inconsistent interpretation, usage, and spelling. In recent decades there has been increasing interest in polyploidy and the word homoeology has experienced an increase in usage. There has been a surge in sequenced plant genomes and polyploid genomes are not far behind, despite their increased complexity and challenges due to their repetitive nature 18, 86.

Thus, just as it was important to establish clear definitions of orthology and paralogy 87, 88, now is the time to establish common and consistent definitions for homologs that exist in a polyploid. Based on our survey of the usage of the term and related concepts in evolutionary biology, we advocate defining homoeologs as pairs of genes that started diverging through speciation but are now found in the same species due to hybridization.

This evolution-based definition has several implications that call for a fundamental shift in the way we as biologists, plant breeders, and bioinformaticians think of homoeology. First, homoeolog inference may suffer from false negatives if inferred solely on the basis of positional conservation. This is because genes can move and, by definition, different types of homologous relationships are based on how the genes originated and not where they are located in the genome. Syntenic conservation is helpful to infer homoeologs but should be used only as a soft criterion to provide additional evidence that a pair of genes are homoeologs. We recommend using the term positional homoeolog when referring to the subset of homoeologs with a conserved syntenic position.

Furthermore, looking at homoeology from an evolutionary perspective has an impact on the **relationship cardinality**. Homoeology is not necessarily a one-to-one relationship, especially in highly duplicated plant genomes. This conceptual change is important because one-to-one positional homoeologs are likely to have significantly different biological characteristics than one-to-many, non-positional homoeologs – as has been previously observed with orthologs.

The establishment of a clear and meaningful definition of homoeology is timely. With rapid progress in sequence technology, we are at the cusp of an explosion of sequenced polyploid genomes. However, although assembling allopolyploid genomes might no longer be ‘formidable’ [18], unraveling the evolutionary history of the genes they contain remains resolutely so (see Outstanding Questions). Overcoming this challenge will require a major coordinated effort among plant, evolutionary, and computational biology scientists. A common definition and framework constitutes a first essential step toward that goal.

Outstanding Questions Can dependable computational methods be devised to infer from genome sequence alone whether a polyploid species originated by allopolyploidization or by autopolyploidization?

Certain computational pipelines need delineation of subgenomes before homoeology inference. This will, however, not work if there has been considerable chromosomal rearrangement between subgenomes after polyploidization. Can one simultaneously detect rearrangement, separate subgenomes, and infer homoeologs?

In general, what are the functional differences between homoeologs (resulting from allopolyploids) and ohnologs (resulting from autopolyploids)? There is a growing body of research looking at the functional implications of polyploidization, but so far a clear answer remains elusive.

## Figures and Tables

**Figure I:**
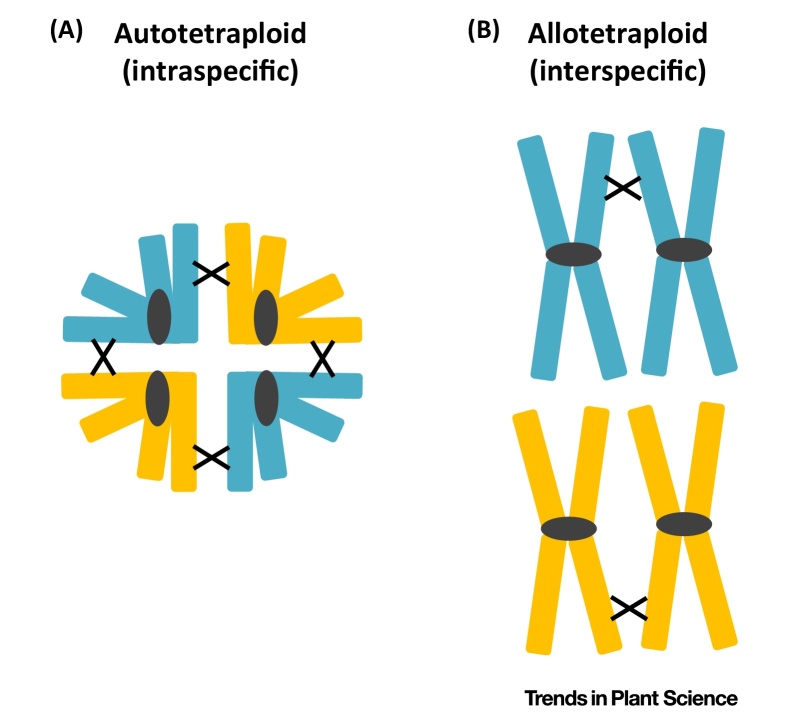
Typical Chromosome Associations during Meiosis in (A) Autopolyploids and (B) Allopolyploids.

**Figure I:**
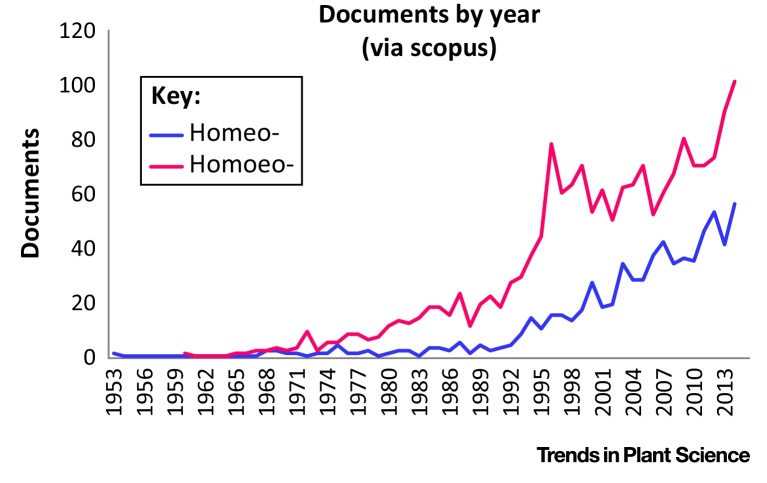
Usage of Homeo- versus Homoeo- in the Literature. A search was performed via Scopus of the primary literature up to the end 2015 and included the search terms homoeology, homoeologous, homoeolog, and homoeologue versus their homeo- forms.

**Table 1:** Varied Usages of the Term ‘Homoeology’ in Different Areas of Research

Context	Definition	Refs
Recombination	Homoeologous: ‘sequences that are similar but imperfectly matched’	[96]
Cytogenetics	Homoeologous chromosomes: ‘those which once were homologous, i.e. essentially identical, but have become so different that they rarely pair [during meiosis]’	[97]
Evolutionary biology	Homoeologous: ‘duplicated genes or chromosomes that are derived from different parental species and are related by ancestry’	[98]
Computational biology	Homoeologs: ‘orthologs between subgenomes’	[35]
This review	Homoeologs: pairs of genes or chromosomes in the same species that originated by speciation and were brought back together in the same genome by allopolyploidization	

## Box 1 Allo- versus Autopolyploidy

*What Are the Types of Polyploidy?*

The criteria for distinguishing and classifying natural polyploids has been subject to a long-standing debate 89, 90. In this review we adopt the most widespread definition, which is based on a taxonomic framework: allopolyploids result from genome doubling following a hybridization between two different species (interspecific), whereas autopolyploids result from genome doubling within one species (intraspecific).  *What Are the Biological Differences between Allo- and Autopolyploids?*

Historically, polyploid types were distinguished by their chromosome pairing behavior observed under the microscope during metaphase I of meiosis [91].

Since autopolyploids are formed by genome doubling within the same species, by consequence autopolyploids originate with an identical set of chromosomes. This means there is an equal opportunity for the homologous chromosomes to pair at meiosis. Thus, autopolyploids are more likely than allopolyploids to form multivalent chromosome configurations – the association of three or more chromosomes during the first meiotic division (Figure IA).

By contrast, allopolyploids usually form bivalent chromosome associations during meiosis. Allopolyploids are derived from different species; thus, the chromosome sets have begun to diverge before the hybridization event. The chromosomes are non-identical and this is one reason why there is a tendency for homologous chromosomes to pair over homoeologous chromosomes, resulting in diploid-like pairing behavior (Figure IB).  *Caveats and Risks of Using Chromosome Pairing to Define Homoeologous Chromosomes*

Distinguishing autopolyploids from allopolyploids based on chromosome pairing has proved to be inadequate [92]. It is impossible to make phylogenetic inferences or statements on homology based on chromosome pairing because pairing behavior is not exact, with many exceptions to the rule. Pairing is at least partially under genetic control, is influenced by the environment, and can be observed between homoeologous and non-homoeologous chromosomes 93, 94, 95.

## Box 2 Alternative Spellings of Homoeology

To make homoeology even more confusing, there are alternative spellings that exist in the literature. The original spelling by Huskins [7] uses an ‘œ’ diphthong borrowed from Latin. This ‘œ’ has been transliterated in modern usage to the ‘oe’ in ‘homoeolog’. However, the alternative spelling ‘homeolog’ has also been used extensively.

Which spelling is more popular? Based on our survey of the literature, homoeolog and its derivatives has 1779 mentions, while homeolog has just 738 mentions (Figure I).

Thus, since it is the most common spelling, we recommend retention of the original homoeology spelling. Regarding pronunciation, it is more difficult to gauge usage across the community, but the Merriam-Webster medical dictionary pronounces homoeologous as ‘ho-mee-o-log-ous’ (http://www.merriam-webster.com/medical/homoeologous). Thus, conveniently, the two alternative spellings are pronounced in the same way.

## Box 3 Challenges of Computational Techniques

There are several challenges shared by similarity- and evolution-based computational approaches, which suffer many of the same challenges as inferring orthologs. For example, errors in sequence assembly can cause problems. Most draft genome sequences have errors in the number of genes due to fragmented assemblies, repetitive regions, low coverage, and propagation of bad gene annotations 99, 100. This becomes a problem particularly in plant species, which often have large, repetitive genomes [101]. Additionally, plant genomes are complex, with large gene families. Paralogs may be merged into chimeric contigs, resulting in incorrect annotations.

Missing genes can pose a problem for homoeolog inference. Missing genes in one subgenome or differential gene loss could cause paralogs that were duplicated before the speciation event to appear as homoeologs. This could be a problem with low-coverage draft genomes [102] or with too-stringent gene prediction methods.

Perhaps the biggest problem with draft genome assemblies for homoeolog inference is not missing genes but fragmented genes, where genes are only partially represented in the sequence. Gaps in sequence give rise to many small contigs, which in turn result in fragmented gene predictions over several contigs. These fragments appear as multiple genes but are actually one gene [103]. Fragmentation causes an overestimation of genes and this could cause overestimation of the number of genes with one-to-many and many-to-many orthology. Additionally, fragmentation makes it harder for algorithms relying on a minimum length of the sequence overlap between homoeologs.

In summary, just as ‘low-quality assemblies result in low-quality annotations’ [99], low-quality annotations result in low-quality homoeology inference.

## Glossary

Allopolyploidy: polyploidy originating from interspecific hybridization followed by genome doubling.
Autopolyploidy: polyploidy originating from intraspecific genome doubling.
Cohomoeologs: set of genes in a subgenome that are all homoeologous to the same genes in another subgenome, thus resulting from subgenome-specific duplications (i.e., duplications that have occurred after speciation of the progenitors).
Comparative mapping: a technique that uses molecular mapping to show conservation of gene order along the chromosomes of related species.
Coorthologs: set of genes found in a species that are all orthologous to the same genes in another species, thus resulting from lineage-specific duplications (i.e., duplications that have occurred after the speciation of the two species in question).
Homoeologs: genes or chromosomes in the same species that originated by speciation and were brought back together in the same genome by allopolyploidization.
Homologous: genes or chromosomes related by common ancestry.
Ohnologs: genes or chromosomes in the same species that originated by a whole-genome duplication event (autopolyploidy).
Orthologs: genes or chromosomes in different species that originated by a speciation event.
Paleolog: genes or chromosomes in the same species that resulted from an ancient polyploidization event.
Paralogs: genes that originated by a duplication event.
Positional homoeologs: homoeologs that have remained in the same position as they were in the progenitor common ancestor.
Relationship cardinality: the number of instances in one entity related to the number of instances in the other (e.g., one-to-one, one-to-many, many-to-many).
Subgenome: one of the genomes in an allopolyploid, each derived from different progenitor species.
